# Multigenerational toxicity of perfluorooctanoic acid (PFOA) on the demography of *Simocephalus vetulus* (Branchiopoda)

**DOI:** 10.1007/s10646-026-03079-8

**Published:** 2026-04-24

**Authors:** Cesar Alejandro Zamora-Barrios, Francisco José Torner-Morales, Rosa Martha Moreno-Gutiérrez, Brendali Zamora-Vargas, Ruth Silvana Cortés-Lagunes, Ricardo Iván Cruz-Cano

**Affiliations:** 1 Laboratory of Water Pollutants Removal Processes, Division of Research and Postgraduate Studies, Universidad Nacional Autónoma de México, FES-Iztacala, State of Mexico, Tlalnepantla, 54090 Mexico; 2 Laboratory of Environmental Microecology, Division of Research and Postgraduate Studies, Universidad Nacional Autónoma de México, FES-Iztacala, Tlalnepantla, 54090 Mexico; 3 Instituto de Ciencias Aplicadas y Tecnología, UNAM Circuito Exterior S/N, Ciudad Universitaria, Coyoacán, Ciudad de México, 04510 México

**Keywords:** Aquatic Toxicology, synthetic pollutant, zooplankton, multigenerational toxicity, life table

## Abstract

Perfluorooctanoic acid (PFOA) is a synthetic pollutant highly persistent in the environment due to its long degradation times. Despite increasingly restricted production, this compound has been extensively used in manufacturing across a wide variety of industries, such as coatings, firefighting foams, synthetic fabrics, packaging, cookware, upholstery, and personal care products since the 1940s. Cladocerans are highly sensitive to environmental stress factors, and these effects are easily observed through alterations in demographic parameters. This sensitivity makes them an effective tool for assessing pollutants. In this study, we assessed the multigenerational effects of PFOA on the survival and fecundity of the cladoceran *Simocephalus vetulus*. The organisms were exposed to sublethal concentrations over five consecutive generations, specifically at levels of 44, 89, and 178 µg L^− 1^, established based on the LC50 of 356 µg L^− 1^. The results showed a decline in all demographic variables across generations, especially at higher concentrations. With 44 µg L^− 1^ PFOA, individuals showed partial recovery in the F1 generation; however, in the F4 generation, life expectancy was reduced by more than 80% compared to control. At concentrations of 89 and 178 µg L^− 1^, survival and reproduction rates declined from the F2 generation onwards, suggesting cumulative toxic effects. Reproduction was inhibited at all concentrations tested. These findings indicate that PFOA exposure compromises the long-term viability of *S. vetulus* populations, with potential implications for aquatic food webs and ecosystem stability.

**Supplementary Information:**

The online version contains supplementary material available at 10.1007/s10646-026-03079-8.

## Introduction

Perfluoroalkyl substances (PFAS) are a large group of synthetic chemicals produced industrially since the 1940s. Initially designed for military usage, PFAS were later adapted for industrial and commercial applications due to their exceptional thermal stability, chemical resistance, and surfactant properties (Haimbaugh et al. 2022; Dong et al. 2025). Among them, perfluorooctanoic acid (PFOA) has received particular attention because of its widespread use and environmental persistence (Wee and Aris 2023). The stable carbon–fluorine bonds confer high resistance to thermal, chemical, and biological degradation (Liu et al. 2016), resulting in an estimated environmental half-life exceeding several decades (over 90 years) and justifying its classification as a “forever chemical” ( Wee and Aris 2023). PFOA is now a ubiquitous toxicant, transported by atmospheric and precipitation processes, and detected even in remote areas, recorded in several abiotic media, including surface water, soil, sediments, groundwater, air, and ice (Soltanian et al. 2024; Arulananthan et al. 2025).

The Stockholm Convention has imposed restrictions on the production of PFOA; however, PFOA continues to be manufactured in developing countries. Reported concentrations in freshwater systems worldwide generally range from ng to µg L^− 1^, as documented in surface and drinking water studies across North America, Europe, and Oceania (Fang et al. 2020; Razak et al. 2023). In China, one of the major producers of PFAS, PFOA concentrations have commonly been found between 3 and 39 µg L^− 1^, with the highest levels reaching 341 µg L^− 1^ in areas affected by fluorochemical manufacturing activities ( Wang et al. 2022; Wee and Aris 2023). A notable hotspot is the Xiaoqing River estuary in China, showing concentrations reached 1.71 mg L^− 1^, the highest level reported to date in surface waters (Liu et al. 2016). PFOA pollution is not limited to highly industrialized countries; for instance, in Mexico, Rodríguez-Varela et al. (2021) found 0.6 µg L^− 1^ in treated wastewater, in the Tula River, and in the Mezquital Valley irrigation canal.

From a toxicological perspective, due to its structural similarity to octanoic acid, PFOA acts as an agonist of the peroxisome proliferator-activated receptor alpha (PPAR*α*), a key signalling pathway involved in its toxicity (Evans et al. 2022). PFOA accumulates primarily in the liver, kidneys, and serum (van den Heuvel et al. 1991), and it induces hepatotoxicity, genotoxicity, immunotoxicity, and neurotoxicity (DeWitt et al. 2008; Dehghani et al. 2025). Its potential for bioaccumulation and trophic biomagnification poses a risk to aquatic fauna, with evidence of adverse effects at low concentrations (Du et al. 2021; Gaines 2023).

Cladocerans functionally transfer energy from phytoplankton to higher trophic levels. Their short generation times, widespread distribution, ease of handling in the laboratory, and high sensitivity to environmental changes make them valuable bioassay organisms in ecotoxicology (Declerck and De Senerpont Domis 2023).

Toxicology research traditionally focuses on short term single generation studies (OECD 211), which limits their ability to uncover multigenerational effects across consecutive generations (F1, F2, F3, etc.), for instance cumulative or inherited effects (Barata et al. 2017; Arreguin-Rebolledo et al. 2023). Maternal pre-exposure can influence offspring tolerance, leading to either positive effects that offer adaptive advantages in early generations or negative effects when chemical stress surpasses compensatory mechanisms and accumulates over time (LaMontagne and McCauley 2001; Zamora-Barrios et al. 2024).

PFOA has multiple toxic effects on freshwater zooplankton at both individual and population levels. Exposure reduces body weight and size, delays reproduction, and lowers offspring production, thereby reducing population density. Chronic exposure disrupts endocrine regulation, increases oxidative stress and DNA damage, and alters behaviors such as swimming and feeding (Yang et al. 2019; Ma et al. 2022). Despite the scarcity of multigenerational ecotoxicity data on PFAS, a few studies have examined these effects in cladocerans. For instance, continuous multigenerational exposure of *Daphnia magna* to PFOS affected fitness parameters in early generations; however, population growth recovered partially in later generations (Jeong et al. 2016). Recent work shows that short-chain PFAS, like PFBS and its precursor FBSA, are more toxic over generations. This is due to the accumulation and transfer of these compounds (Xie et al. 2025). Furthermore, PFOA, when present alongside other emerging contaminants, can induce synergistic effects, while biological responses may be modulated by prior environmental exposure (Soltanighias et al. 2024).

Tropical and subtropical waterbodies are dominated by cladoceran species, primarily those belonging to the genera *Diaphanosoma*, *Moina*, *Ceriodaphnia*, *Chydorus*, *Alona*, *Macrothrix*, and *Simocephalus* (Nandini and Sarma 2023). Despite this, *D. magna*, a Holarctic species considered exotic in tropical areas, is still widely used in accredited laboratories for toxicity testing (Tkaczyk et al. 2021; Zamora-Barrios et al. 2025). The selection of native species versus standard organisms for bioassays should align with the specific objectives of the study. Sentinel cladocerans such as *D. magna* facilitate cross-study comparisons and establish a globally consistent framework for ecotoxicological assessment. In contrast, native species are valuable for evaluating ecological risks within local environmental contexts (Rodgher et al. 2010). *Simocephalus vetulus* is one of the largest daphnids inhabiting tropical and subtropical ecosystems and is widely regarded as a reliable bioindicator, due to its sensitivity to environmental xenobiotics (Nguyen et al. 2024). Evaluations that incorporate this specie demonstrates that, as a locally abundant native taxon, it provides a more realistic representation of toxicant effects (Santos-Medrano and Rico-Martínez 2019).

Here, we have evaluated the multigenerational ecotoxicity of PFOA on the cladoceran *S. vetulus*. Organisms were exposed to sublethal concentrations equivalent to 12.5, 25, and 50% of the LC50 for five consecutive generations. Demographic parameters, including survival, fertility, lifespan, generation time, and population growth rate, were assessed. We tested two alternative hypotheses: (H1) a positive maternal effect, in which prior exposure leads to adaptive responses that mitigate impacts on early generations, and (H2) cumulative toxicity without adaptation, resulting in a gradual decline in population viability. This investigation provides new evidence regarding the long-term ecological risks associated with PFOA and highlights the importance of incorporating multigenerational perspectives into environmental risk assessments.

## Materials and methods

### Laboratory maintenance of *Simocephalus vetulus*

The cladoceran populations used in the experimental treatments were taxonomically identified as *S. vetulus* based on specialized taxonomic keys (Korovochinsky and Smirnov 1998). *S. vetulus* is considered a species complex; therefore, it is treated here as a morphospecies. A monoclonal lineage was established from a single parthenogenetic female isolated from the littoral zone of Lake Zumpango, Mexico (19° 48′ N and 99° 06′ W). Stock cultures were derived from this founder individual and maintained under controlled laboratory conditions for over five years. Cultures were kept in 1 L glass beakers filled with synthetic, moderately hard reconstituted freshwater prepared according to U.S. Environmental Protection Agency (EPA) guidelines (reconstituted by dissolving 0.002 g L^− 1^ KCl, 0.06 g MgSO₄, 0.06 g L^− 1^ CaSO₄, and 0.095 g L^− 1^ NaHCO₃ in distilled water). The medium was renewed twice per week. Cultures were maintained at 22 ± 2 °C under natural photoperiod (12:12 h light-dark) and fed every three days with *Chlorella vulgaris* at a concentration of 0.5 × 10^6^ cells mL^− 1^ as the sole food source.

### Microalgal culture (*Chlorella vulgaris*)

*Chlorella vulgaris* (strain CL-V-3, CICESE, Mexico) was cultivated in 2 L graduated Erlenmeyer flasks containing sterile Bold’s basal medium (Borowitzka and Borowitzka 1988). To enhance carbon availability, 0.5 g of NaHCO_3_ was added every three days. Cultures were inoculated at an initial density of 1 × 10^5^ cells mL^− 1^ and maintained at 25 ± 2 °C under a 16:8 h light-dark photoperiod with an irradiance of 150 µmol m^− 1^ s^− 1^. Moderate aeration was applied to prevent sedimentation, using 0.22 μm filters at the inlet and outlet to avoid bacterial contamination. Algal cells were harvested during the exponential growth phase, decanted, and centrifuged at 2,500 rpm to remove residual medium. The pellet was resuspended, and cell density was quantified using a Neubauer hemocytometer.

### Toxicant Preparation

Perfluorooctanoic acid (CAS No. 335-67-1) was the tested toxicant. The compound was obtained in crystal form (5 g presentation, batch #171468) from Sigma-Aldrich, Spruce Street, St. Louis, MO 63,103, USA (distributed by Merck), with a purity of 95%. Stock solutions (1 g L^− 1^) were prepared using ultrapure water and stored at 4 °C in amber glass bottles to prevent degradation or photolysis.

## Analytical determination of PFOA in liquid media

The determination of PFOA in water samples was performed using a high-performance liquid chromatography system (HPLC, 1260 Infinity Series, Agilent Technologies) coupled to an electrospray ionization (ESI) source and a triple quadrupole mass spectrometer (6420, Agilent Technologies). To minimize background PFAS contamination in chromatographic analysis, polytetrafluoroethylene (PTFE) solvent inlet filters and the purge valve were removed from the HPLC system and replaced with stainless steel components.

All the liquid samples were filtered using a 0.45 μm membrane, and 10 µL aliquots were injected in the HPLCS-MS/MS system. The chromatographic separation was achieved using a Zorbax SB-C18 column (150 mm × 3.0 mm, 3.5 μm particle size), with a mobile phase consisted of 10 mM ammonium acetate in ultrapure water (A) and acetonitrile (B), delivered at a constant flow rate of 0.3 mL min^− 1^ under isocratic conditions. The retention time of PFOA was approximately 3.8 min, with a total run time of 8 min per injection.

Mass spectrometry detection was conducted using negative electrospray ionization mode. Nitrogen was used as the drying gas at 300 °C, with a flow rate of 11 L min^− 1^. The nebulizer pressure was set to 15 psi, and the capillary voltage was maintained at 1000 V. The precursor ion was monitored at m/z = 412.9, while product ions were detected at m/z = 269 and 169. The fragmentor voltage was set to 65 V and the collision energy to 3 eV. The acceleration cell voltage was 2 V.

Quantification was performed using matrix-matched calibration curves consisting of 16 concentration levels covering a total dynamic range from 0.01 to 4 mg L^− 1^. Due to the wide concentration range required for acute and chronic exposure tests, two linear calibration intervals were established: a low-range calibration from 0.01 to 0.4 mg L^− 1^ and a high-range calibration from 0.5 to 4 mg L^− 1^. Calibration curves are presented in Figure S1.

### Acute toxicity assay and dose selection

An acute toxicity assay was conducted solely to determine the sublethal concentrations for the subsequent chronic, multigenerational experiment. To this end, the LC50 of PFOA for the cladoceran was estimated under controlled laboratory conditions. Six nominal concentrations (100, 200, 400, 800, 1600, and 3200 µg L^− 1^) were prepared, along with a toxicant-free control. Acute toxicity tests were performed in borosilicate glass containers filled with 40 mL of each concentration diluted in EPA medium. Each treatment was performed in quadruplicate. For each replicate, 10 neonates (< 24 h old) obtained from the third generation of the stock culture were placed in individual containers, resulting in a total of 40 organisms per concentration. Organisms were incubated for 48 h at 22 ± 2 °C under static conditions, fasting conditions, and underneath 16:8 photoperiod. After the exposure period, mortality (defined as immobility for 30 s after gentle mechanical stimulation) was evaluated using a stereomicroscope.

### Multigenerational chronic assays

**Fig. 1:**
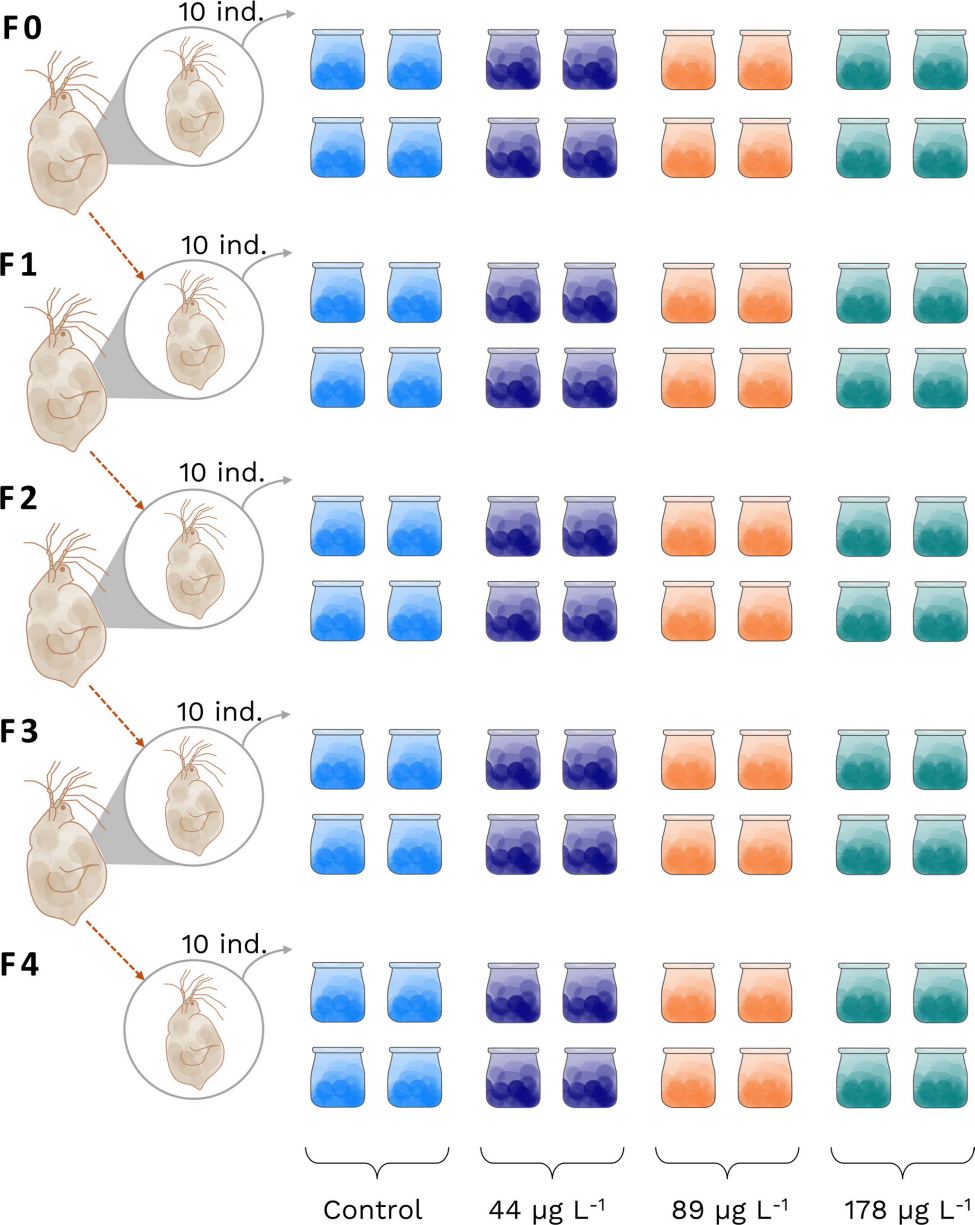
Experimental design of the multigenerational bioassays on *S. vetulus* exposed to PFOA. The parental generation (F0) was initiated with 10 individuals in each treatment (control, 44, 89, and 178 µg L⁻¹). From each cohort, 10 neonates were selected to establish the subsequent filial generation (F1-F4)

Multigenerational chronic assays were conducted to evaluate the effects of PFOA on the population dynamics of *S. vetulus*. The experiment was initiated using neonates (< 24 h old). Each treatment was performed in quadruplicate. For each replicate, 10 organisms were placed individually into 60 mL borosilicate glass vessels containing 40 mL of EPA medium with the corresponding chronic toxicant concentration, for a total of 40 organisms per treatment. These consisted of an uncontaminated control and three concentrations of PFOA (44, 89, and 178 µg L^− 1^, selected based on sublethal fractions of 12.5, 25, and 50% of the determined LC50). Bioassays were designed to evaluate the effects of the toxicant across successive generations (F0, F1, F2, F3, and F4). Multigenerational experiments were carried out using offspring from the first reproductive event of each cohort (Fig. 1). These neonates were collected, and ten individuals were randomly selected to initiate the next generation under the same treatment conditions. The experiments were conducted in an incubator maintained at a temperature of 22 ± 1 °C, with a 16:8-hour light: dark photoperiod. All experimental organisms, regardless of treatment, were fed with *C. vulgaris* at a concentration of 0.5 × 10^6^ cells mL^− 1^. Toxicant exposure was continuous, with the medium being renewed daily. For this, individuals were gently filtered through a 200 μm mesh to remove residual debris and transferred to a fresh medium. Under a stereoscopic microscope, survival, mortality, and the number of offspring per cohort were recorded every 24 h. The experiments were ended when the last individual of each treatment died.

With the recorded data, we calculated survivorship and fecundity and determined life table demographic variables, including average lifespan, net reproduction, reproductive growth, generation time, and population growth (Krebs 1985). In this context, *x* = age; *lₓ=*proportion of individuals surviving to age *x*; *mₓ* = mean number of neonates produced per surviving individual at age x; *eₓ* = life expectancy of organisms living at the beginning of age *x*; Tₓ= cumulative number of individual-days lived from age *x* onward; nₓ= number of individuals alive at the beginning of age *x*. *e* = 2.71828, and r = intrinsic rate of population increases. *R₀* = average number of offspring produced per individual over its lifetime.

Life expectancy:

$$\:{\boldsymbol{e}}_{\boldsymbol{x}}=\frac{{\boldsymbol{T}}_{\boldsymbol{x}}}{{\boldsymbol{n}}_{\boldsymbol{x}}}$$

Gross reproductive rate:

$$\:=\sum\:_{0}^{\boldsymbol{\infty\:}}{\boldsymbol{m}}_{\boldsymbol{x}\boldsymbol{\infty\:}}$$

Net reproductive rate:

$$\:=\sum\:_{0}^{\boldsymbol{\infty\:}}{\boldsymbol{l}}_{\boldsymbol{x}}{\boldsymbol{m}}_{\boldsymbol{x}}$$

Generation time:

$$\:\boldsymbol{T}\:=\frac{\sum\:{\boldsymbol{l}}_{\boldsymbol{x}}{\boldsymbol{m}}_{\boldsymbol{x}}\boldsymbol{x}}{{\boldsymbol{R}}_{0}}$$

Rate of population increase (*r*), solved iteratively:

$$\:\sum\:_{\boldsymbol{x}=\boldsymbol{w}}^{\boldsymbol{n}}{\boldsymbol{e}}^{-\boldsymbol{r}\boldsymbol{x}}{\boldsymbol{l}}_{\boldsymbol{x}}{\boldsymbol{m}}_{\boldsymbol{x}}=1$$

Statistical analyses were conducted using SigmaPlot 11.0. The median lethal concentration was determined by interpolating the concentration corresponding to a probit value of 5, indicating a 50% mortality rate. This statistical approach enables the estimation of toxicity thresholds based on dose-response correlation (Finney 1971). Survival outcomes were evaluated using the Kaplan-Meier method, and statistical significance was determined with a *post hoc* Holm-Sidak analysis. Significant differences in life table parameters were assessed using one-way ANOVA followed by Tukey’s *post hoc* test. A Two-way ANOVA was employed to determine the effects of PFOA concentration and generation, as well as their interaction, on demographic variables.

## Results

### PFOA concentrations

Analytical verification of PFOA used in bioassays displayed close agreement between nominal values and measured values in both acute and chronic exposure experiments. In the LC50 assay, measured concentrations differed only slightly from theoretical values, with deviations ranging from 2.1 to 8.2% across the tested concentration range (100 to 3200 µg L^− 1^, Table S1). For the chronic exposure assays, measured concentrations in freshly prepared media were also consistent with nominal targets. The low (44 µg L^− 1^), medium (89 µg L^− 1^), and high (178 µg L^− 1^) treatments showed measured initial concentrations of 44.99, 93.33, and 178.55 µg L^−1^, respectively (Table S2).

### Acute toxicity response of *Simocephalus vetulus* to PFOA

**Fig. 2:**
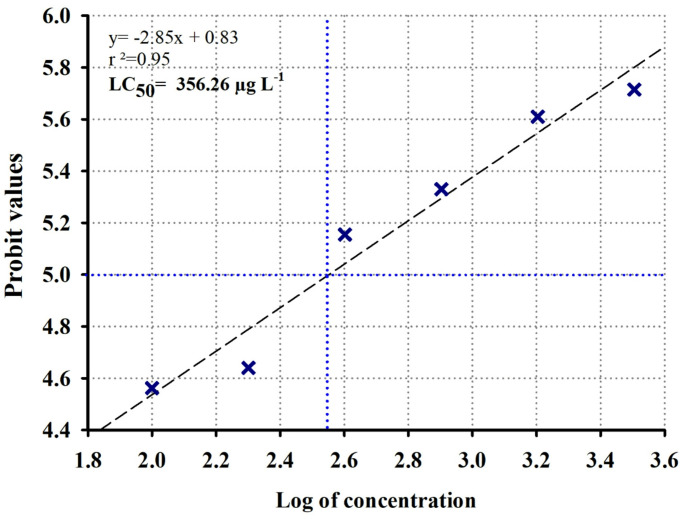
LC50 of *S. vetulus* determined after exposure to PFOA at six concentrations. The x-axis represents the logarithm of the concentrations (100, 200, 400, 800, 1600, and 3200 µg L^− 1^), while the y-axis corresponds to the probit-transformed mortality values

The LC50 estimated for *S. vetulus* exposed to PFOA was 356.26 µg L^− 1^ ± 1.96 (95% CI: 150.96-561.58; Fig. 2). Based on this value, three sublethal concentrations of 44, 89, and 178 µg L^− 1^, were selected to evaluate the multigenerational effects across five generations (F0 to F4).

### Survivorship across generations

**Fig. 3:**
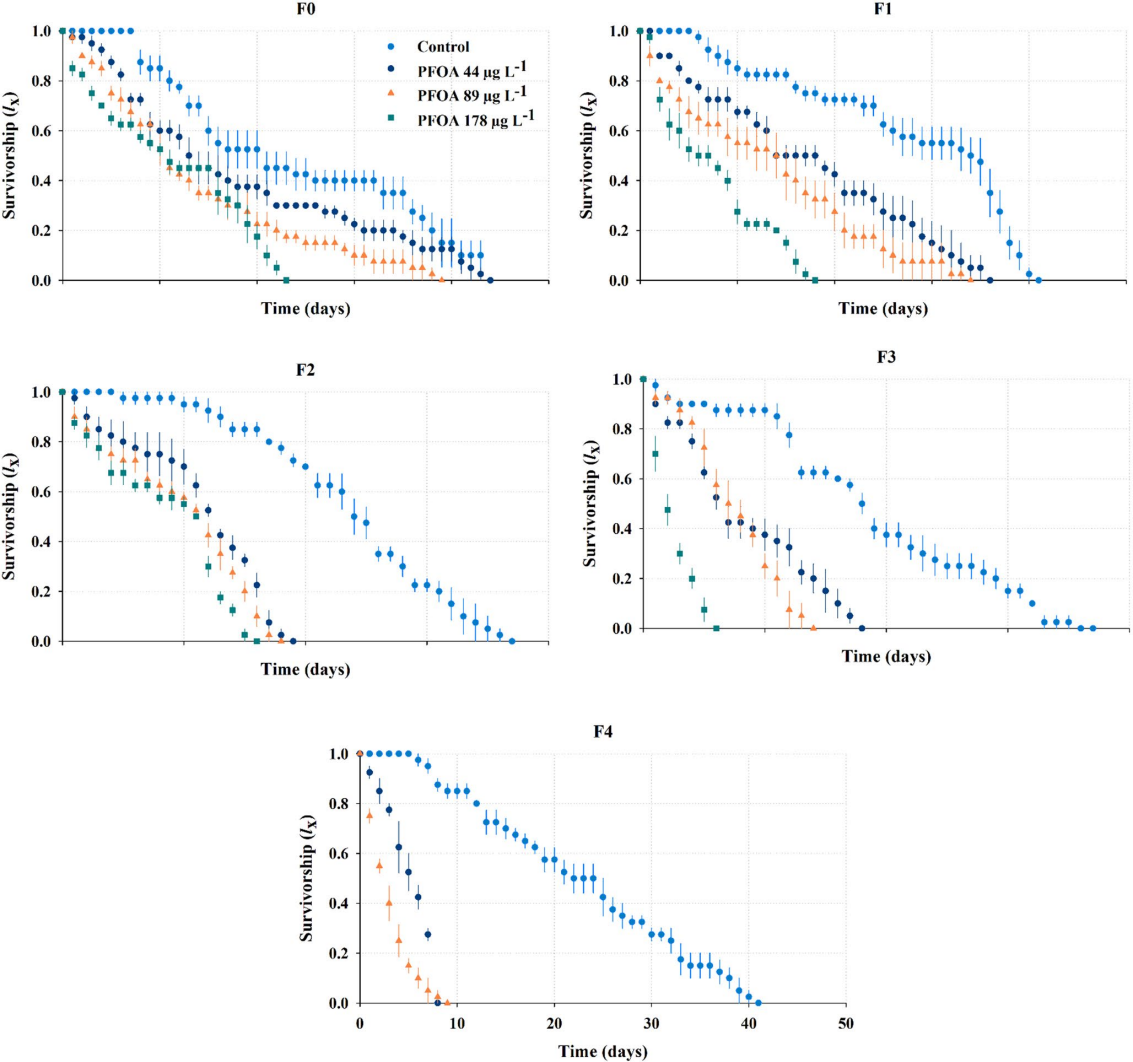
Survivorship curves of *S. vetulus* across five successive generations (F0-F4) under chronic exposure to perfluorooctanoic acid (44, 89, and 178 µg L^− 1^). Values are presented as mean ± SE from four replicates

PFOA significantly reduced survivorship across successive generations, with generational and dose-dependent patterns. In the control group, survivorship remained stable across all generations. Mean of maximum survival times ranged from 32.8 ± 0.75 to 40.5 ± 1.44 days, with no significant differences among generations (Fig. 3; Table 1, Kaplan-Meier survival analysis, Holm-Sidak *post hoc* test, P *<* 0.05,). The F0 generation exhibited the maximum observed survival (43 days). At 44 µg L^− 1^, survivorship in F0 (39.8 ± 1.70) and F1 (33.8 ± 2.06) did not differ significantly from the control (Kaplan-Meier survival analysis, Holm-Sidak *post hoc* test, P *<* 0.05). In later generations, mean survival declined to 16.8 ± 0.48 days in F2 and 15.5 ± 0.87 days in F3. In F4, mean survival collapsed to 7 days (Table 1). At 89 µg L^− 1^, the mean maximum survival decreased significantly by approximately 20%, from 33.0 ± 1.83 days in F0 to 26.8 ± 2.14 days in F1 (P *<* 0.05). Severe reductions were observed in F3 (11.5 ± 0.50 days; ~65%) and F4 (6.3 ± 0.63 days; ~81%) (Table 1; Fig. 3). At the highest concentration (178 µg L^− 1^), survivorship was significantly reduced in all generations compared to the control (Holm-Sidak *post hoc* test P *<* 0.05). Survival decreased from 21.3 ± 0.48 days in F0 to 15.3 ± 0.25 days in F1, 14.3 ± 0.25 days in F2, and reached only 4.5 ± 0.29 days in F3, being the treatment with the greatest reduction observed (Fig. 3).

**Table 1:** Maximum observed survival (± SE) and median survival time for each generation and treatment. Different letters indicate significant differences within each generation (Kaplan-Meier Survival Analysis, Holm-Sidak, *P* < 0.05)

Generation	Treatment (µg L⁻¹)	Mean survival (days ± SE)	Median (days)	Group
F0	Control	40.5 ± 1.44	38	a
	44	39.8 ± 1.70	40	a
	89	33.0 ± 1.83	31	b
	178	21.3 ± 0.48	21	c
F1	Control	36.3 ± 2.18	37	a
	44	33.8 ± 2.06	31	a
	89	26.8 ± 2.14	24	ab
	178	15.3 ± 0.25	15	b
F2	Control	33.3 ± 1.03	33	a
	44	16.8 ± 0.48	16	b
	89	16.3 ± 0.48	16	b
	178	14.3 ± 0.25	14	c
F3	Control	32.8 ± 0.75	32	a
	44	15.5 ± 0.87	14	b
	89	11.5 ± 0.50	11	c
	178	4.5 ± 0.29	4	d
F4	Control	37.0 ± 1.73	38	a
	44	7.0 ± 0.00	7	b
	89	6.3 ± 0.63	6	b

### Fecundity across generations

**Fig. 4:**
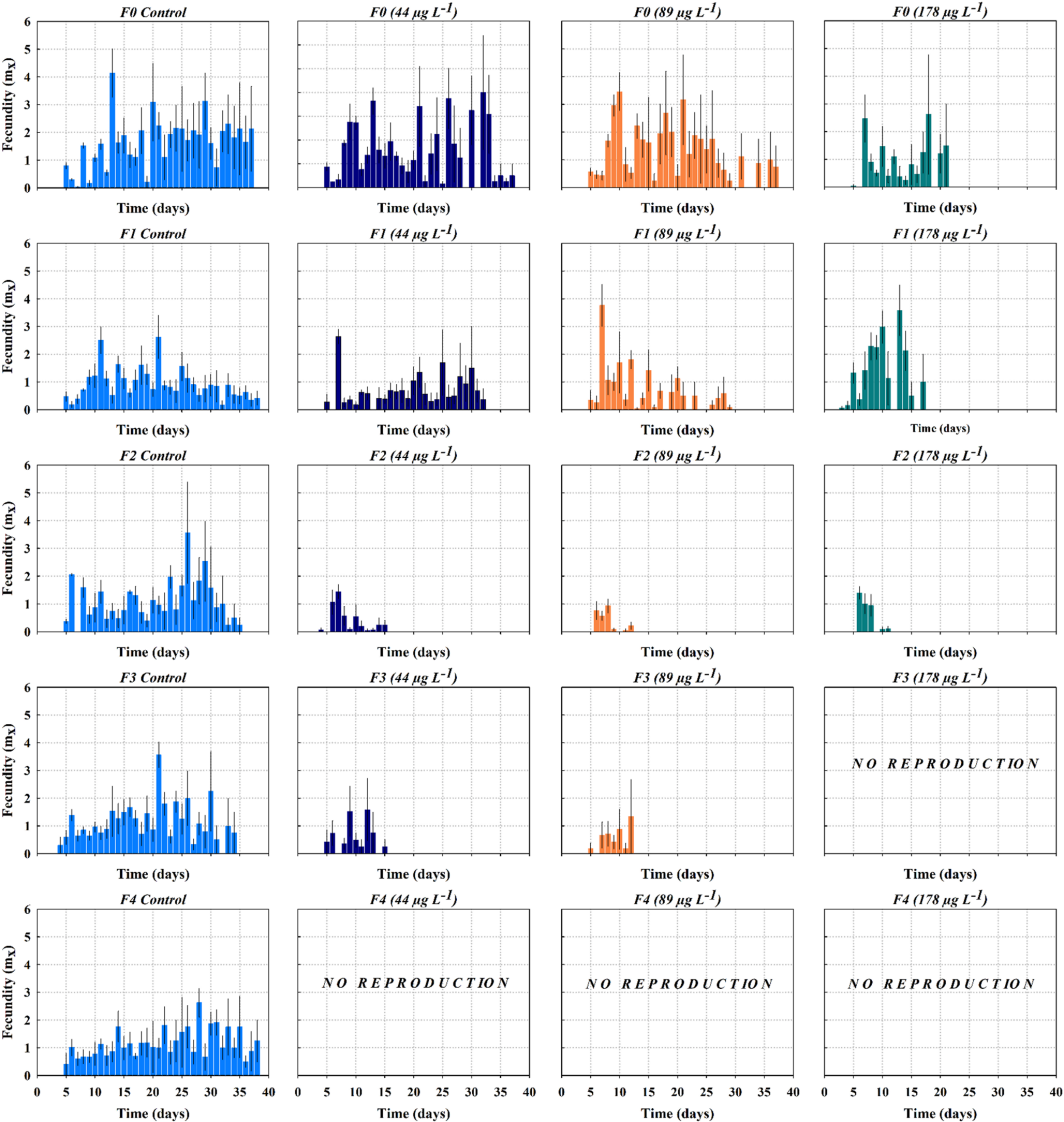
Fecundity of *S.vetulus* across five successive generations (F0-F4) under chronic exposure to perfluorooctanoic acid (44, 89, and 178 µg L^− 1^). Values are presented as mean ± SE from four replicates

In the control treatment, all generations (F0-F4) exhibited continuous reproductive activity throughout the experimental period, with no generational decline in reproductive performance observed. Reproduction consistently began between days 5 and 6 in all generations. The highest daily fecundity occurred in F0 on day 13 (4.14 ind^− 1^ d^− 1^), with similarly elevated peaks in subsequent generations (Fig. 4). The duration of reproductive activity was consistent among generations, lasting 33 days in F0, 34 days in F1 and F4, and approximately 30 days in F2 and F3, with reproduction typically persisting until days 35–38. Exposure to 44 µg L^− 1^ of PFOA resulted in reproductive performance in the F0 and F1 generations comparable to that of the control group in terms of onset (days 5–6), peak fecundity (often exceeding 2.0 ind^− 1^ d^− 1^), and total duration Beginning with the F2 generation, reproductive output declined significantly, with reproduction occurring over a shorter, earlier time interval, reduced daily peak values, and earlier cessation (approximately days 12–15). At F3 generation, reproductive activity was sporadic and limited to low-magnitude peaks, culminating in complete reproductive inhibition in the F4 generation (Fig. 4). At the intermedial concentration evaluated (89 µg L^− 1^), reproductive activity declined more rapidly and at earlier stages across generations. The F0 maintained reproduction for approximately 29 days, with a maximum fecundity of 3.46 neonates ind^− 1^ d^− 1^. In subsequent generations (F2 generation), reproduction was primarily limited to the first half of the life cycle, with a maximum value of 0.93 ind^− 1^ d^− 1^ on day 8 (Fig. 4). In F3 and F4, reproductive output was minimal and short-lived, with *mx* values declining rapidly to zero shortly after reproduction began. At the highest concentration tested (178 µg L^− 1^), reproductive activity was severely restricted across generations. In F0 and F1, reproduction lasted approximately 14 days, representing an estimated 60% reduction in reproductive period compared to the control. Daily fecundity values were low and highly variable, with only a few isolated reproductive events. In F1, reproduction began prematurely around day 4. Maximum fertility reached values of up to ~ 3.6 ind^− 1^ d^− 1^, concentrated in a brief time interval. By F3 and F4 generations, reproductive activity was entirely suppressed.

### Life-table variables across generations

#### Average life span

**Fig. 5:**
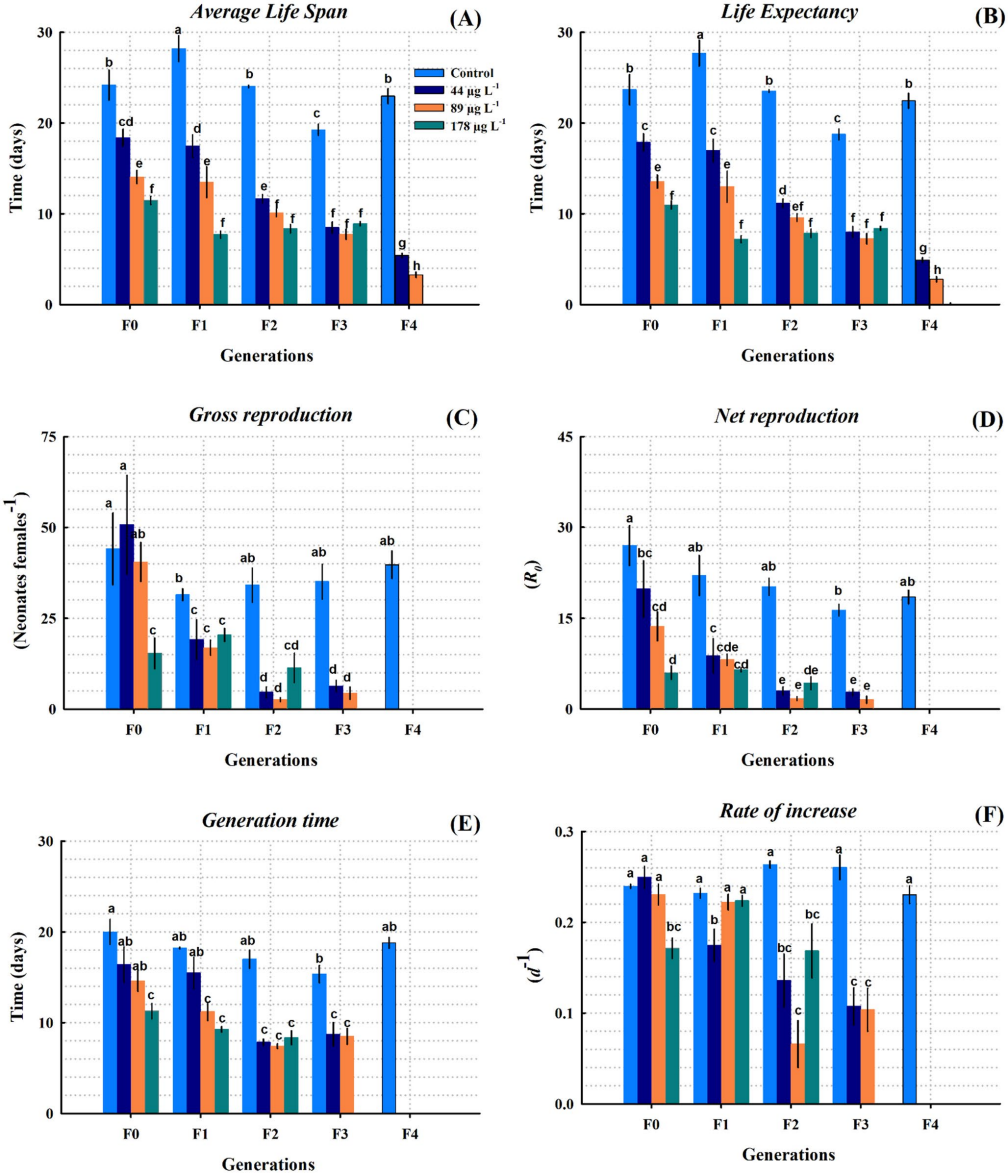
Demographic life table variables of *S. vetulus* exposed to 44, 89, and 178 µg L^− 1^ of PFOA across five successive generations (F0-F4). Different letters indicate significant differences between treatments according to the *post hoc* Tukey test (*P* < 0.05)

Average life span (ALS) of *S. vetulus* was significantly influenced by PFOA concentration (Table 2, two-way ANOVA, F = 269.140, *P* < 0.001) and generation (F = 53.218, *P* < 0.001). A significant interaction between treatment and generation showed that the variable decreased across generations (two-way ANOVA, *P* < 0.001). In the control, ALS ranged from 19.2 to 28.2 days, with the last value being the highest across all experiments (F0-F4) and observed in the F2 generation. In F1, all treatments differed significantly (*P* < 0.05). As concentration increased, average life span (ALS) decreased by 24% (18.3 days at 44 µg L^− 1^), 41% (14.05 days at 89 µg L^− 1^), and 52% (11.4 days at 178 µg L^− 1^) compared to the control (Fig. 5A). In F3, the control group had an ALS of 19.25 ± 0.61 days, significantly longer than in all exposed groups (Table 3, one-way ANOVA, *P* < 0.05). Organisms exposed to 44, 89, and 178 µg L^− 1^ had mean life spans of 8.48 ± 0.62, 7.75 ± 0.57, and 8.90 ± 0.23 days, respectively, representing a 54–60% reduction relative to the control. No significant differences were observed among the toxic tested concentrations (*P* > 0.05). In F4, the ALS could not be assessed at 178 µg L^− 1^ due to population collapse (Fig. 5A). The control group had an ALS of 22.95 ± 0.83 days, while those exposed to 44 and 89 µg L^− 1^ had significantly lower values (5.40 ± 0.25 and 3.28 ± 0.30 days), corresponding to reductions of 76.5 and 85.7% compared to the control (one-way ANOVA, *P* < 0.05). 

**Table 2:** Results of the two-way ANOVA applied to the demographic life table variables of *S. vetulus* exposed to PFOA at three concentrations (44, 89, and 178 µg L^− 1^) across five successive generations (F0-F4)

Source of Variation	DF	SS	MS	F	*P*
**Average life span**					
Treatment	3	3108.235	1036.078	269.140	< 0.001
Generation	4	819.465	204.866	53.218	< 0.001
Treatment × Generation	12	256.816	21.401	5.559	< 0.001
Residual	60	230.976	3.850		
**Gross reproduction**					
Treatment	3	3000.907	1000.302	259.846	< 0.001
Generation	4	819.465	204.866	53.218	< 0.001
Treatment × Generation	12	256.816	21.401	5.559	< 0.001
Residual	60	230.976	3.850		
**Net reproduction**					
Treatment	3	3798.856	1266.285	108.798	< 0.001
Generation	4	1608.317	402.079	34.546	< 0.001
Treatment × Generation	12	342.399	28.533	2.452	0.011
Residual	60	698.332	11.639		
**Generational time**					
Treatment	3	1664.884	554.961	97.455	< 0.001
Generation	4	1157.494	289.374	50.816	< 0.001
Treatment × Generation	12	397.669	33.139	5.819	< 0.001
Residual	60	341.671	5.695		
**Population increases**					
Treatment	3	0.317	0.106	43.155	< 0.001
Generation	4	0.354	0.0884	36.115	< 0.001
Treatment × Generation	12	0.195	0.0163	6.657	< 0.001
Residual	60	0.147	0.00245		

Factors considered were treatment (concentration) and generation. DF = degrees of freedom; SS = sum of squares; MS = mean square; F = Fisher’s test; P = significance value

**Table 3:** Results of the one-way ANOVA applied to the demographic life table variables of *S. vetulus* exposed to PFOA at three concentrations (44, 89, and 178 µg L^− 1^) and the control across five successive generations (F0-F4)

Source of Variation	DF	SS		MS	F	*P*
**Average Life Span**						
Between Groups	19	4188.868		220.467	80.925	< 0.001
Residual	60	163.460		2.724		
Total	79	4352.328				
**Life Expectancy**						
Between Groups	19	4081.568		214.819	78.852	< 0.001
Residual	60	163.460		2.724		
Total	79	4245.028				
Between Groups	19	4081.568		214.819		
**Gross reproduction**						
Between Groups	19	24545.338		1291.860	14.250	< 0.001
Residual	60	5439.392		90.657		
Total	79	29984.730				
**Net reproduction**						
Between Groups	19	5786.133		304.533	26.459	< 0.001
Residual	60	690.585		11.510		
Total	79	6476.718				
**Generation time**						
Between Groups	19	3329.176		175.220	50.943	< 0.001
Residual	60	206.372		3.440		
**Rate of increase**						
Between Groups	19	0.888		0.0468	20.620	< 0.001
Residual	60	0.136		0.00227		
Total	79	1.024				

DF = degrees of freedom; SS = sum of squares; MS = mean square; F = Fisher’s test; P = significance value

### Life expectancy

Life expectancy at birth (LF) in the F0 generation showed that the control had a value of 23.68 ± 1.65 days, significantly higher than that of all the treatments exposed (*P* < 0.05). Exposure to 44, 89, and 178 µg L^− 1^ reduced this variable to 17.88 ± 0.95, 13.55 ± 0.73, and 10.98 ± 0.44 days, respectively, compared to the control. At the lowest concentration (44 µg L^− 1^), LF did not differ significantly between the F0 and F1 generations (*P* > 0.05), with values of 17.88 ± 0.95 and 16.95 ± 1.25 days, respectively (Fig. 5B). However, from generation F2 onward, a significant decrease was observed (*P* < 0.05); LF dropped to 11.15 ± 0.47 days in F2, 7.98 ± 0.62 days in F3, and 4.90 ± 0.25 days in F4. This demonstrates a cumulative transgenerational effect, even at the lowest concentration tested. By the F4 generation, the control showed an LF of 22.45 ± 0.83 days, whereas exposure to 44 µg L^− 1^ significantly reduced it to 4.90 ± 0.25 days (*P* < 0.05). At 89 µg L^− 1^, the reduction was more pronounced, with a value of 2.78 ± 0.30 days, corresponding to an approximately 88% decrease relative to the control (*P* < 0.05).

### Gross reproduction rate

The gross reproduction rate (GRR) was significantly affected by treatment, generation, and their interaction (two-way ANOVA, *P* < 0.001). In generation F0, GRR values at 44 and 89 µg L^− 1^ were 50.75 ± 13.61 and 40.43 ± 5.38 neonates female^− 1^, respectively, both statistically similar to the control (44.10 ± 9.90 neonates female^− 1^; Tukey, *P* > 0.05). In contrast, exposure to 178 µg L^− 1^ resulted in a 65% reduction in GRR (15.37 ± 4.23 neonates female⁻¹) (Fig. 5C). In generation F1, GRR of *S. vetulus* decreased significantly across all exposed treatments (Tukey, *P* < 0.05), with reductions of 39% (44 µg L^− 1^), 46% (89 µg L^− 1^), and 35% (178 µg L^− 1^) compared with the control (31.46 ± 1.64 neonates female^− 1^). In generation F2, severe reproductive decrease was observed at 89 µg L^− 1^, with reductions of 92% (2.63 ± 0.52), relative to the control (34.09 ± 4.68 neonates female^− 1^). From generation F3 onward, reproductive inhibition became pronounced, with reductions of 82% (44 µg L^− 1^) and 88% (89 µg L^− 1^) compared with the control, while reproduction was completely inhibited at 178 µg L^− 1^ (Tukey, *P* < 0.05). In generation F4, GRR was suppressed in the remaining toxic treatments (see Fig. 5C).

### Net reproductive rate

In PFOA-free populations of *S. vetulus*, net reproductive values (*R₀*) remained stable across generations, ranging from 16.33 to 26.98 neonate females^− 1^, with an overall generational mean of 20.79 neonate females^− 1^ (Fig. 5D). In contrast, exposure to 44 µg L^− 1^ of PFOA caused a statistical decrease in *R₀* in the F1 generation (8.80 ± 2.84 neonate females^− 1^), (Tukey, *P* < 0.05). This decrease progressed through subsequent generations, falling to 2.98 ± 0.67 in F2 and 2.75 ± 0.56 neonate females^− 1^ in F3, until *R₀* was completely suppressed in F4. At 89 µg L^− 1^, *R₀* decreased from 13.68 ± 2.39 neonate females^− 1^ in F0 to 8.15 ± 0.95 in F1 and 1.70 ± 0.31 in F2, reaching 1.53 ± 0.59 neonate females^− 1^ in F3. These values correspond to reductions of 50–90% relative to the control in that generation (Tukey, *P* < 0.05, Fig. 5D). At the highest concentration (178 µg L^− 1^), reproductive impairment was evident from the first generation, with a reduction in *R₀* to 6.00 ± 1.09 neonate females^− 1^ (~ 78% reduction).

### Generation time

Chronic exposure to PFOA significantly reduced the generation time (GT) of *S. vetulus*, with the magnitude of reduction dependent on both concentration and generation (Two-way ANOVA, *P* < 0.001). In the F0 generation, GT values were 16.40 ± 1.93 days at 44 µg L^− 1^, 14.60 ± 1.14 days at 89 µg L^− 1^, and 11.27 ± 0.86 days at 178 µg L^− 1^, all shorter than the control (19.99 ± 1.38 days). In F1, GT further decreased to 15.47 ± 1.73 days at 44 µg L^− 1^, 11.19 ± 0.98 days at 89 µg L^− 1^, and 9.26 ± 0.31 days at 178 µg L^− 1^, each lower than the control (18.21 ± 0.10 days), representing reductions of 15–49%. In F2, all PFOA treatments resulted in shorter GTs: 7.83 ± 0.35 days (44 µg L^− 1^), 7.41 ± 0.25 days (89 µg L^− 1^), and 8.34 ± 0.76 days (178 µg L^− 1^), each significantly shorter than the control (16.99 ± 0.99 days; Tukey, *P* < 0.05). The F3 generation exhibited similar trends, with GTs of 8.74 ± 1.28 and 8.49 ± 0.88 days, respectively (Tukey, *P* < 0.05). In F4, *S. vetulus* populations collapsed in all PFOA treatments, whereas the control maintained a generation time of 18.79 ± 0.60 days (Fig. 5E).

### Intrinsic rate of increase

In the PFOA-free treatment, the intrinsic rate of increase (*r*) remained stable across generations, with values ranging from 0.23 to 0.26 d^− 1^, and no significant differences among cohorts (Tukey, *P* > 0.05) (Fig. 5F). In the F0 generation, cladocerans exposed to 44 and 89 µg L^− 1^ of PFOA showed growth rates values of 0.25 ± 0.01 and 0.23 ± 0.01 d^− 1^, respectively. There were no significant differences compared to the control (Tukey, *P* > 0.05). In contrast, exposure to 178 µg L^− 1^ significantly reduced *r* (0.17 ± 0.01 d^− 1^). In the following F1 generation, *r* decreased to 0.17 ± 0.02 d^− 1^ in the 44 µg L^− 1^ treatment, about 25% lower than the control (Tukey, *P* < 0.05). At 89 and 178 µg L^− 1^, *r* values (0.22 ± 0.01 and 0.22 ± 0.01 d^− 1^) stayed comparable to the control. In the F2 generation, *S. vetulus* populations differed between treatments. The control had the highest *r* value (0.26 ± 0.004 d^− 1^). At 44 µg L^− 1^, *r* dropped significantly (0.14 ± 0.03 d^− 1^). The decrease was more pronounced at 89 µg L^− 1^, where *r* fell to 0.07 ± 0.03 d^− 1^, about a 75% reduction versus the control. At 178 µg L^− 1^, population growth was statistically similar to the previous generation (0.17 ± 0.03 d^− 1^; Tukey, *P* < 0.05). Advancing into the F3 generation, *r* values in all exposed treatments were significantly lower than in the control, but statistically similar between treatments (Tukey, *P* < 0.05), with values of 0.11 ± 0.02 d^− 1^ at 44 µg L^− 1^ and 0.10 ± 0.02 d^− 1^ at 89 µg L^− 1^. Notably, at 178 µg L^− 1^ concentration, complete demographic failure was observed, a similar pattern was observed in the F4 generation, where population growth collapsed in treatments containing 44 and 89 µg L^− 1^ of PFOA (Fig. 5F).

## Discussion

The estimated acute lethal concentration (LC50) for *S. vetulus* populations in this study (356 µg L^− 1^) exceeds the concentrations typically reported in freshwater aquaculture systems, which are generally in the order of ng L^− 1^. Nevertheless, PFAS contamination levels can range from µg L^− 1^ to mg L^− 1^ in systems affected by industrial effluents (Liu et al. 2016; Lee et al. 2020). In this context, the acute concentrations used in the present study represent extreme exposure scenarios, which should be considered when extrapolating these results to broader environmental contexts. On the other hand, the chronic concentrations evaluated in this study were generally higher than those typically reported in natural freshwater systems, with the exception of the lowest treatment (~ 40 µg L^− 1^), which falls within the range of PFOA concentrations documented in inland waters from industrialized regions (Wang et al. 2022). The remaining concentrations represent elevated exposure levels that are more likely to occur at critical contamination hotspots, particularly in areas receiving direct industrial discharges. Consequently, the results obtained here should be interpreted as reflecting worst-case exposure scenarios and provide valuable insight into the potential multigenerational effects of PFOA under highly contaminated conditions.

Previous studies have demonstrated wide interspecific variation in the acute toxicity of PFOA to cladocerans. For instance, Barmentlo et al. (2015) reported a sublethal effect (EC50: immobilization) of 239 mg L^− 1^ for *D. magna*, indicating a tolerance nearly 670 times higher than that of *S. vetulus* in our evaluation. These findings reinforce the idea that holarctic cladocerans are not ideal for laboratory assessments in tropical or subtropical regions, where they are not native and often exhibit greater tolerance than local species (Espinoza-Rodríguez et al. 2024; Zamora-Barrios et al. 2025). It was previously proved by Razak et al. (2023) who showed that the tropical cladoceran *Moina micrura* exhibited a LC50 for PFOA of 474.7 µg L^− 1^, which is comparable in magnitude to the estimated here for *S. vetulus*. This comparison supports the high sensitivity of tropical cladocerans to PFOA and reinforces the ecological relevance of our findings.

Consistent with this variability, differences have also been documented within the genus *Simocephalus*. Morehouse et al. (2025) reported higher tolerance to PFOA in *S. serrulatus*, with LC50 values of 261–272 mg L^− 1^, which are several orders of magnitude higher than those observed for *S. vetulus* in the present study. This discrepancy likely reflects genuine species-specific variation. Methodological factors, including organism age (Sarma and Nandini 2006) and feeding prior to exposure, also influence the sensitivity of exposed organisms (Zamora-Barrios et al. 2026), as these conditions alter physiological status and the absorption of toxic substances. These methodological differences may partly explain the contradictory results and highlight that sensitivity to PFAS cannot be generalized across an entire genus.

The U.S. EPA water quality criteria for PFOA in freshwater ecosystems include acute and chronic thresholds of 3.1 mg L^− 1^ and 100 µg L^− 1^, respectively (U.S. EPA 2024). Our findings show that *S. vetulus* had an LC50 approximately 8.7 times lower than the acute threshold and of the same order of magnitude as the chronic threshold, including values both below and above this level (2.27 and 1.12 times lower, and 1.78 times higher, respectively), highlighting the remarkable sensitivity of this tropical cladoceran. Importantly, demographic and multigenerational effects were observed even at concentrations below the chronic threshold (44 and 89 µg L^− 1^), suggesting that regulatory values may underestimate risks to non-model species under realistic exposure conditions. von der Ohe and Liess (2004) developed an interspecies sensitivity index that enables comparisons between the sensitivity of aquatic invertebrates versus *D*. *magna*, the globally accepted sentinel species. According to this approach, a relative sensitivity value of zero indicates equivalent sensitivity, while positive values denote lower sensitivity and negative values reflect higher sensitivity. In this context, *S. vetulus* could be one of the most sensitive cladoceran species recorded (Table 4). 

**Table 4:** RS values were calculated as log (LC_50 *D. magna*_/LC_50*i*_ or EC_50*i*_), where LC_50*D. magna*_ corresponds to the most tolerant *D. magna* strain (675,000 µg L^− 1^), and LC_50*i*_ or EC_50*i*_ refers to the experimental LC_50_ of species *i*. Higher RS values indicate greater sensitivity relative to the reference species

Species	Assay Type	Concentration (µg L^− 1^)	CAS No.	RS	Reference
*S. vetulus*	LC50	356	335-67-1	3.2779	This study
*M. micrura*	LC50	475	335-67-1	3.1526	Razak et al. (2023)
*D. carinata*	EC50	78,200	335-67-1	0.9361	Logeshwaran et al. (2021)
*D. magna*	EC50	80,300	335-67-1	0.9246	Lu et al. (2015)
*C. sphaericus*	EC50	91,100	335-67-1	0.8698	Ding et al. (2012)
*D. magna*	LC50	137,000	335-67-1	0.6926	Verhille and Hausler (2024)
*M. macrocopa*	EC50	200,000	335-67-1	0.5283	Ji et al. (2008)
*D. pulicaria*	EC50	203,700	335-67-1	0.5203	Boudreau (2009)
*D. magna*	EC50	223,600	335-67-1	0.4798	Boudreau (2009)
*D. magna*	EC50	239,000	335-67-1	0.4509	Barmentlo et al. (2015)
*D. magna*	EC50	220,000	335-67-1	0.4869	Ding et al. (2012)
*S. serrulatus*	LC50	272,000	335-67-1	0.3741	Morehouse et al. (2025)
*D. magna*	EC50	480,000	382526-1	0.1481	Colombo et al. (2008)
*D. magna*	EC50	632,000	335-95-5	0.0286	Sanderson et al. (2004)
*D. magna*	LC50	675,000	335-67-1	-	Ji et al. (2008)

CAS numbers correspond to the specific chemical form of PFOA tested in each study: acid form (CAS 335-67-1), anionic form (CAS 45285-51-6), ammonium salt (CAS 3825-26-1), and sodium salt provided as an aqueous solution by 3 M Company (CAS 335-95-5)

Our findings indicate that chronic exposure to PFOA progressively undermines the survival of *S. vetulus* across multiple generations, exhibiting a clear dose- and generation-dependent pattern. In both the F0 and F1 generations of *S. vetulus*, survival at low concentrations (44 and 89 µg L^− 1^) remained comparable to those in control groups; nevertheless, continued exposure significantly accelerated the decline in viability. This observation aligns with the results reported by Ji et al. (2008), who noted similar multigenerational effects in *D. magna* and *M. macrocopa*, as well as in a fish model (*Danio rerio*), where Haimbaugh et al. (2022) found transcriptional dysregulation in mitochondrial, endocrine, and immune pathways across generations. Additionally, while biochemical alterations have been observed at the individual level, including neurodegeneration, immune suppression, and oxidative stress, these changes are reflected in the reduced body size of individuals in subsequent generations (Yang et al. 2019). However, compensation based on maternal effects under exposure to PFOS and PFOA has been reported, favouring survival; however, these assessments were limited to consecutive generations (F0 to F1), masking cumulative or transgenerational effects (Jeong et al. 2016; Seyoum et al. 2020). Recent evidence suggests that PFOA toxicity is significantly increased by microplastics, which increase bioavailability and internal exposure. Soltanighias et al. (2024) reported that PFOA alone impaired key fitness-related traits in *Daphnia magna*, including reduced growth and delayed development, without mortality. However, co-exposure with PET microplastics resulted in both additive and synergistic effects on ecological endpoints.

The collapse of reproduction in the F3 generation at the highest concentration, and in the F4 generation across other treatments, suggests the accumulation and intergenerational transfer of pollutants. This delayed reproductive impairment aligns with multigenerational toxicity studies in cladocerans, where severe effects often appear only after several generations of continuous exposure, even if earlier generations show tolerance or partial recovery (Padilla Suárez et al. 2023). Xie et al. (2025) investigated the effects of perfluorobutanesulfonic acid (PFBS) on *Daphnia magna* and observed a multigenerational reproductive decline of ~ 45% from the parental to F3. This decline was linked to progressive bioaccumulation and the surpassing of internal toxicity thresholds. Upon reaching this threshold, physiological resistance diminished, resulting in further bioaccumulation in subsequent generations and adverse impacts on both reproduction and population growth. In later generations (F4 to F5), reproductive capacity was entirely lost. In contrast, Jeong et al. (2016) found that PFOS exposure reduced offspring per clutch in *D. magna* from the F0 to F2 generations, but adverse effects lessened in later generations, indicating partial recovery of fecundity under certain exposure and re-exposure conditions.

At the mechanistic level, reproductive collapse observed at all tested concentrations may be associated with disruptions in physiological and endocrine processes. These findings are consistent with broader evidence indicating that PFOA acts as an endocrine disruptor, directly interfering with hormonal pathways crucial for egg production and embryonic development (Pezeshki et al. 2025). Comparable responses in other members of zooplankton have been reported in the rotifer *Brachionus calyciflorus*, where chronic PFOA exposure altered fecundity, shifted the proportion of mictic females, and reduced resting egg production (Zhang et al. 2014). Also, PFAS have been shown to induce nuclear fragmentation in earthworms without causing immediate lethality. This effect is associated with oxidative stress, DNA damage, reduced antioxidant capacity, and hepatic accumulation (Pietropoli et al. 2025). Future studies that compare the mechanisms underlying sensitivity to PFAS could help clarify these interspecific differences; in particular, more species should be analyzed to test whether the trend observed here occurs across a wide variety of taxa.

The life expectancy of *S. vetulus* under optimal conditions ranges from 20 to 30 days (Nandini et al. 2000). Our results are consistent with these reports (20.2–28.2 days); however, this cladoceran showed a decrease that was dependent on concentration under PFOA exposure, with reductions of up to 70% in F1 generation at the highest concentration tested. This severe effect is comparable to that observed with other xenobiotic compounds. For instance, Nguyen et al. (2024) reported that exposure to delafloxacin reduced life expectancy by 35 to 40% in the F0 and F1 generations, while affecting other demographic traits such as age-specific fecundity and population growth rate.

Exposure to sublethal concentrations of PFOA significantly diminished the growth rate of the *S. vetulus* population and altered the generation time. Shorter generation times are associated with higher intrinsic population growth rates under optimal conditions (Cole 1954). However, studies on zooplankton show that reductions in this demographic variable are also associated with stress-induced responses. When exposed to stressors such as pollutants, cladocerans may exhibit earlier maturation and reproduction, but this is often offset by shorter longevity and lower total reproductive output (Hulot et al. 2012; Zamora-Barrios et al. 2025). These trade-offs often result in a decrease in population growth rate, indicating physiological deterioration rather than increased fitness (Grzesiuk et al. 2024).

Populations of *S. vetulus* exposed to higher concentrations of PFOA consistently exhibited lower intrinsic growth rates (*r*). A decrease in the r serves as an integrative indicator of stress at the population level, as it reflects alterations in survival, reproduction, and generation time. Chronic exposure to perfluoroalkyl and polyfluoroalkyl substances (PFAS) alters the metabolic and physiological processes of aquatic organisms including cladocerans, leading to decreased reproductive output and impaired population growth. Taken together, these responses underscore the usefulness of *r* as a sensitive indicator of the long-term toxicity of PFAS (Hong et al. 2025). Recently, research by Xie et al. (2025) revealed that the *r* in *D. magna* was progressively affected across generations when subjected to perfluorobutane sulfonic acid (PFBS) and its precursor, perfluorobutane sulfonamide (FBSA). Reductions of 63.7, 37.4, and 14.9% were recorded in the F1, F3, and F4 generations, respectively. Although there was a partial recovery in adult survival at later stages, the population’s growth capacity continued to be significantly compromised, ultimately resulting in extinction by the F5 generation. Similar reductions of *r* have been observed in rotifers chronically exposed to both PFOA and PFOS, with reproductive disruption being the primary factor underlying the fitness decline (Zhang et al. 2013, 2014).

Evidence indicates that PFAS, particularly PFOA, destabilize zooplankton population parameters. Chronic exposure to these xenobiotic compounds has common consequences, such as decreased fertility, reduced longevity, and diminished population growth rates (Zhang et al. 2014; Bartlett et al. 2021). These findings highlight the value of functional demographic parameters as early and sensitive indicators of ecological risk, as they capture responses inherent to chemical stress. At the community level, exposure to PFOA has been linked to significant declines in sensitive taxa and alterations in zooplankton community composition (Sanderson et al. 2003), confirming that the effects extend beyond individual organisms. In the long term, the persistence and accumulation of PFAS increase the possibility of population collapse and biodiversity loss, which could have knock-on effects on trophic dynamics and the stability of freshwater ecosystems (Ma et al. 2022).

## Conclusion

Acute and chronic exposure to PFOA had detrimental effects on the survival and reproductive success of *Simocephalus vetulus*, highlighting the vulnerability even at low concentrations. The multigenerational effects, characterised by decreased survival and reproductive inhibition, underscore the potential risks to cladoceran populations in PFAS-contaminated environments, with potentially greater impacts in tropical ecosystems. This study emphasises the need to reassess regulatory thresholds, as continuous or recurrent exposure to PFOA may pose significant ecological risks, suggesting that even levels considered safe could cause a severe decline in cladoceran populations over time.

## Supplementary Information

Below is the link to the electronic supplementary material.

Supplementary Material 1

## Data Availability

No datasets were generated or analysed during the current study.

## References

[CR1] Arreguín-Rebolledo U, Páez-Osuna F, Betancourt-Lozano M, Rico-Martínez R (2023) Multi- and transgenerational synergistic effects of glyphosate and chlorpyrifos at environmentally relevant concentrations in the estuarine rotifer *Proales similis*. Environ Pollut 318:120708. 10.1016/j.envpol.2022.120708 36410595 10.1016/j.envpol.2022.120708

[CR2] Arulananthan A, Vilhelmsson OÞ, Karsten U, Grossart HP, Sigurbjörnsdóttir A, Rolfsson Ó, Scholz B (2025) Per- and polyfluoroalkyl substances (PFAS) in the cryosphere – occurrence, organismic accumulation, ecotoxicological impacts, transformation, and management strategies. Front Environ Sci 13:1559941. 10.3389/fenvs.2025.1559941

[CR3] Barata C, Campos B, Rivetti C, LeBlanc GA, Eytcheson S, McKnight S, De Schamphelaere K (2017) Validation of a two-generational reproduction test in *Daphnia magna*: an interlaboratory exercise. Sci Total Environ 579:1073–1083. 10.1016/j.scitotenv.2016.11.101 27908627 10.1016/j.scitotenv.2016.11.066 PMC5488698

[CR4] Barmentlo SH, Stel JM, van Doorn M, Eschauzier C, de Voogt P, Kraak MH (2015) Acute and chronic toxicity of short chained perfluoroalkyl substances to *Daphnia magna*. Environ Pollut 198:47–53. 10.1016/j.envpol.2014.12.007 25553346 10.1016/j.envpol.2014.12.025

[CR5] Bartlett AJ, De Silva AO, Schissler DM, Hedges AM, Brown LR, Shires K, Miller J, Sullivan C, Spencer C, Parrott JL (2021) Lethal and sublethal toxicity of perfluorooctanoic acid (PFOA) in chronic tests with *Hyalella azteca* (amphipod) and early-life stage tests with *Pimephales promelas* (fathead minnow). Ecotoxicol Environ Saf 207:111250. 10.1016/j.ecoenv.2020.111250 32920311 10.1016/j.ecoenv.2020.111250

[CR67] Borowitzka MA, Borowitzka LJ (1988) Micro-algal biotechnology. Cambridge University Press, Cambridge

[CR7] Boudreau TM (2009) *Toxicity of perfluorinated organic acids to selected freshwater organisms under laboratory and semifield conditions.* Master thesis, University of Guelph, Environmental Biology, Ontario, Canada

[CR9] Cole LC (1954) The population consequences of life history phenomena. Q Rev Biol 29:103–137. 10.1086/400074 13177850 10.1086/400074

[CR8] Colombo I, de Wolf W, Thompson RS, Farrar DG, Hoke RA, L’Haridon J (2008) Acute and chronic aquatic toxicity of ammonium perfluorooctanoate (APFO) to freshwater organisms. Ecotoxicol Environ Saf 71(3):749–756. 10.1016/j.ecoenv.2008.01.003 18538392 10.1016/j.ecoenv.2008.04.002

[CR10] Declerck SA, de Senerpont Domis LN (2023) Contribution of freshwater metazooplankton to aquatic ecosystem services: an overview. Hydrobiologia 850:2795–2810. 10.1007/s10750-023-05278-3

[CR11] Dehghani MH, Aghaei M, Bashardoust P, Rezvani Ghalhari M, Nayeri D, Malekpoor M, Shi Z (2025) An insight into the environmental and human health impacts of per- and polyfluoroalkyl substances (PFAS): exploring exposure pathways and their implications. Environ Sci Eur 37:81. 10.1186/s12302-025-01091-4

[CR12] DeWitt JC, Copeland CB, Strynar MJ, Luebke RW (2008) Perfluorooctanoic acid–induced immunomodulation in adult C57BL/6J or C57BL/6 N female mice. Environ Health Perspect 116(5):644–650 18470313 10.1289/ehp.10896 PMC2367677

[CR13] Ding GH, Frömel T, van den Brandhof EJ, Baerselman R, Peijnenburg WJ (2012) Acute toxicity of poly- and perfluorinated compounds to two cladocerans, *Daphnia magna* and *Chydorus sphaericus*. Environ Toxicol Chem 31:605–610. 10.1002/etc.1720 22170568 10.1002/etc.1713

[CR15] Dong Z, Ji G, Wang F, Wang F (2025) Per- and polyfluoroalkyl substances (PFAS): Current prevalence, regulatory frameworks, and safe drinking water guidelines in the United States. J Environ Chem Eng 118145. 10.1016/j.jece.2025.118145

[CR14] Du D, Lu Y, Zhou Y, Li Q, Zhang M, Han G, Jeppesen E (2021) Bioaccumulation, trophic transfer and biomagnification of perfluoroalkyl acids (PFAAs) in the marine food web of the South China Sea. J Hazard Mater 405:124681. 10.1016/j.jhazmat.2020.124681 33307411 10.1016/j.jhazmat.2020.124681

[CR71] Espinosa-Rodríguez CA, Jiménez-Santos MA, Martínez-Miranda DM, Piedra-Ibarra E, Rivera-De la Parra L, Lugo-Vázquez A (2024) *Daphnia magna* (Crustacea: Anomopoda) in central Mexico wetlands: implications of escape from ecotoxicological laboratories. Biol Invasions 26:1–7 10.1007/s10530-023-03164-7

[CR16] Evans N, Conley JM, Cardon M, Hartig P, Medlock-Kakaley E, Gray LE Jr (2022) *In vitro* activity of a panel of per- and polyfluoroalkyl substances (PFAS), fatty acids, and pharmaceuticals in peroxisome proliferator-activated receptor (PPAR) alpha, PPAR gamma, and estrogen receptor assays. Toxicol Appl Pharmacol 449:116136. 10.1016/j.taap.2022.116136 35752307 10.1016/j.taap.2022.116136 PMC9341220

[CR17] Fang S, Sha B, Yin H, Bian Y, Yuan B, Cousins IT (2020) Environment occurrence of perfluoroalkyl acids and associated human health risks near a major fluorochemical manufacturing park in southwest of China. J Hazard Mater 396:122617. 10.1016/j.jhazmat.2020.122617 32298866 10.1016/j.jhazmat.2020.122617

[CR18] Finney DJ (1971) Probit analysis: a statistical treatment of the sigmoid response curve, 3rd edn. Cambridge University Press, Cambridge

[CR19] Gaines LG (2023) Historical and current usage of per- and polyfluoroalkyl substances (PFAS): A literature review. Am J Ind Med 66:353–378. 10.1002/ajim.23490 35614869 10.1002/ajim.23362

[CR20] Grzesiuk M, Kurek M, Białoskórski T, Świątek Z, Bańkowska-Sobczak A (2024) Chronic exposure to environmental contaminants reduces population growth rate and fitness-related traits in *Daphnia magna*. Environ Sci Pollut Res 31:35045–35056. 10.1007/s11356-024-35045-4

[CR21] Haimbaugh A, Wu CC, Akemann C, Meyer DN, Connell M, Abdi M, Khalaf A, Johnson D, Baker TR (2022) Multi- and transgenerational effects of developmental exposure to environmental levels of PFAS and PFAS mixture in zebrafish (*Danio rerio*). Toxics 10:334. 10.3390/toxics10060334 35736942 10.3390/toxics10060334 PMC9228135

[CR23] Hong MS, Lee JS, Lee MC, Lee JS (2025) Ecotoxicological effects of per- and polyfluoroalkyl substances in aquatic organisms: a review. Mar Pollut Bull 214:117678. 10.1016/j.marpolbul.2025.117678 39983440 10.1016/j.marpolbul.2025.117678

[CR22] Hulot FD, Carmignac D, Legendre S, Yepremian C, Bernard C (2012) Effects of microcystin-producing and microcystin-free strains of *Planktothrix agardhii* on long-term population dynamics of *Daphnia magna*. Ann Limnol Int J Limnol 48:337–347. 10.1051/limn/2012023

[CR24] Jeong TY, Yuk MS, Jeon J, Kim SD (2016) Multigenerational effect of perfluorooctane sulfonate (PFOS) on the individual fitness and population growth of *Daphnia magna*. Sci Total Environ 569:1553–1560. 10.1016/j.scitotenv.2016.06.243 27396314 10.1016/j.scitotenv.2016.06.249

[CR25] Ji K, Kim Y, Oh S, Ahn B, Jo H, Choi K (2008) Toxicity of perfluorooctane sulfonic acid and perfluorooctanoic acid on freshwater macroinvertebrates (*Daphnia magna* and *Moina macrocopa*) and fish (*Oryzias latipes*). Environ Toxicol Chem 27:2159–2168. 10.1897/08-123.1 18593212 10.1897/07-523.1

[CR70] Korovochinsky N, Smirnov N (1998) Introduction to the Cladocera (Ctenopoda, Anomopoda, Onychopoda and Haplopoda). Supplemented for America. AN Severtsov Institute of Animal Evolutionary Morphology and Ecology of the Russian Academy of Sciences

[CR26] Krebs CJ (1985) Ecology: the experimental analysis of distribution and abundance. Harper & Row, New York

[CR27] LaMontagne JM, McCauley E (2001) Maternal effects in *Daphnia*: what mothers are telling their offspring and do they listen? Ecol Lett 4:64–71. 10.1046/j.1461-0248.2001.00198.x

[CR28] Lee YM, Lee JY, Kim MK, Yang H, Lee JE, Son Y, Zoh KD (2020) Concentration and distribution of per- and polyfluoroalkyl substances (PFAS) in the Asan Lake area of South Korea. J Hazard Mater 381:120909. 10.1016/j.jhazmat.2019.120909 31352148 10.1016/j.jhazmat.2019.120909

[CR29] Liu Z, Lu Y, Wang T, Wang P, Li Q, Johnson AC, Sarvajayakesavalu S, Sweetman AJ (2016) Risk assessment and source identification of perfluoroalkyl acids in surface and ground water: spatial distribution around a mega-fluorochemical industrial park, China. Environ Int 91:69–77. 10.1016/j.envint.2016.02.020 26909815 10.1016/j.envint.2016.02.020

[CR30] Logeshwaran P, Sivaram AK, Surapaneni A, Kannan K, Naidu R, Megharaj M (2021) Exposure to perfluorooctanesulfonate (PFOS) but not perfluorooctanoic acid (PFOA) at ppb concentration induces chronic toxicity in *Daphnia carinata*. Sci Total Environ 769:144577. 10.1016/j.scitotenv.2020.144577 33482550 10.1016/j.scitotenv.2020.144577

[CR31] Lu GH, Liu JC, Sun LS, Yuan LJ (2015) Toxicity of perfluorononanoic acid and perfluorooctane sulfonate to *Daphnia magna*. Water Sci Eng 8:40–48. 10.1016/j.wse.2015.02.004

[CR32] Ma T, Ye C, Wang T, Li X, Luo Y (2022) Toxicity of per- and polyfluoroalkyl substances to aquatic invertebrates, planktons, and microorganisms. Int J Environ Res Public Health 19:16729. 10.3390/ijerph192416729 36554610 10.3390/ijerph192416729 PMC9779086

[CR34] Morehouse JD, Jones DK, Choi Y, Lee LS, Hoverman JT (2025) Disparities in per- and polyfluoroalkyl substances (PFAS) tolerance and life history traits in *Simocephalus serrulatus* populations. CLEAN Soil Air Water 53:e70071. 10.1002/clen.70071

[CR35] Nandini S, Sarma SSS (2000) Lifetable demography of four cladoceran species in relation to algal food (*Chlorella vulgaris*) density. Hydrobiologia 435:117–126. 10.1023/A:1004049511682

[CR36] Nandini S, Sarma SSS (2023) Experimental studies on zooplankton-toxic cyanobacteria interactions: a Review. Toxics 11:176. 10.3390/toxics11020176 36851051 10.3390/toxics11020176 PMC9965014

[CR37] Nguyen TD, Itayama T, Tran QV, Dao TS, Iqbal MS, Pham TL (2024) Ecotoxicity of the fluoroquinolone antibiotic delafloxacin to the water flea *Simocephalus vetulus* and its offspring under the influence of calcium modulation. Sci Total Environ 923:171450. 10.1016/j.scitotenv.2024.171450 38438028 10.1016/j.scitotenv.2024.171450

[CR38] Padilla Suarez EG, Pugliese S, Galdiero E, Guida M, Libralato G, Saviano L, Spampinato M, Pappalardo C, Siciliano A (2023) Multigenerational tests on *Daphnia* spp.: a vision and new perspectives. Environ Pollut 337:122629. 10.1016/j.envpol.2023.122629 37775025 10.1016/j.envpol.2023.122629

[CR39] Pezeshki H, Rajabi S, Hashemi M, Moradalizadeh S, Nasab H (2025) Per- and poly-fluoroalkyl substances as forever chemicals in drinking water: Unraveling the nexus with obesity and endocrine disruption – A mini review. Heliyon 11:e42782. 10.1016/j.heliyon.2025.e42782 40066031 10.1016/j.heliyon.2025.e42782 PMC11891678

[CR40] Pietropoli E, Schumann S, Moressa A, Gallocchio F, Zonta G, Santovito G, Irato P (2025) Naturally occurring environmental PFAS mixtures induce significant oxidative damage and nuclei fragmentation in *Dendrobaena veneta*. Chemosphere 378:144413. 10.1016/j.chemosphere.2025.144413 40262334 10.1016/j.chemosphere.2025.144413

[CR41] Razak MR, Aris AZ, Zainuddin AH, Yusoff FM, Yusof ZNB, Kim SD, Kim KW (2023) Acute toxicity and risk assessment of perfluorooctanoic acid (PFOA) and perfluorooctanesulfonate (PFOS) in tropical cladocerans *Moina micrura*. Chemosphere 313:137377. 10.1016/j.chemosphere.2022.137377 36457264 10.1016/j.chemosphere.2022.137377

[CR44] Rodgher S, Espíndola ELG, Lombardi AT (2010) Suitability of *Daphnia similis* as an alternative organism in ecotoxicological tests: implications for metal toxicity. Ecotoxicology 19:1027–1033. 10.1007/s10646-010-0484-1 20306222 10.1007/s10646-010-0484-1

[CR42] Rodríguez-Varela M, Durán-Álvarez JC, Jiménez-Cisneros B, Zamora O, Prado B (2021) Occurrence of perfluorinated carboxylic acids in Mexico City’s wastewater: a monitoring study in the sewerage and a mega wastewater treatment plant. Sci Total Environ 774:145060 10.1016/j.scitotenv.2021.145060 33609836 10.1016/j.scitotenv.2021.145060

[CR45] Sanderson H, Boudreau TM, Mabury SA, Solomon KR (2003) Impact of perfluorooctanoic acid on the structure of the zooplankton community in indoor microcosms. Aquat Toxicol 62:227–234. 10.1016/S0166-445X(02)00186-9 12560171 10.1016/s0166-445x(02)00100-5

[CR46] Sanderson H, Boudreau TM, Mabury SA, Solomon KR (2004) Effects of perfluorooctane sulfonate and perfluorooctanoic acid on the zooplanktonic community. Ecotoxicol Environ Saf 58:68–76. 10.1016/S0147-6513(03)00105-9 15087165 10.1016/j.ecoenv.2003.09.012

[CR47] Santos-Medrano GE, Rico-Martínez R (2019) Acute sensitivity comparison among *Daphnia magna* Straus, 1820, *Daphnia pulex* Leydig, 1860 and *Simocephalus vetulus* Müller, 1776, exposed to nine toxicants. Turk J Fish Aquat Sci 19:615–623. 10.4194/1303-2712-v19_7_05

[CR48] Sarma SSS, Nandini S (2006) Review of recent ecotoxicological studies on cladocerans. J Environ Sci Health B 41:1417–1430. 10.1080/03601230600964316 17090502 10.1080/03601230600964316

[CR74] Seyoum A, Pradhan A, Jass J, Olsson PE (2020) Perfluorinated alkyl substances impede growth, reproduction, lipid metabolism and lifespan in Daphnia magna. Sci Total Environ 737:139682. 10.1016/j.scitotenv.2020.139682 32521362 10.1016/j.scitotenv.2020.139682

[CR50] Soltanian M, Gitipour S, Baghdadi M, Rtimi S (2024) PFOA-contaminated soil remediation: a comprehensive review. Environ Sci Pollut Res 31:49985–50011. 10.1007/s11356-024-33812-4 10.1007/s11356-024-34516-y 39088169

[CR49] Soltanighias T, Umar A, Abdullahi M, Abdallah MAE, Orsini L (2024) Combined toxicity of perfluoroalkyl substances and microplastics on the sentinel species *Daphnia magna*: implications for freshwater ecosystems. Environ Pollut 363:125133. 10.1016/j.envpol.2024.125133 39419463 10.1016/j.envpol.2024.125133

[CR51] Tkaczyk A, Bownik A, Dudka J, Kowal K, Ślaska B (2021) *Daphnia magna* model in the toxicity assessment of pharmaceuticals: a review. Sci Total Environ 763:143038. 10.1016/j.scitotenv.2020.143038 33127157 10.1016/j.scitotenv.2020.143038

[CR72] U.S. Environmental Protection Agency (2024) Final freshwater aquatic life ambient water quality criteria and acute saltwater benchmark for perfluorooctanoic acid (PFOA). Office of Water, EPA-842-R-24-002 https://www.epa.gov/system/files/documents/2024-09/pfoa-report-2024.pdf

[CR53] van Heuvel JP, Kuslikis BI, van Rafelghem MJ, Peterson RE (1991) Tissue distribution, metabolism, and elimination of perfluorooctanoic acid in male and female rats. J Biochem Toxicol 6:83–92. 10.1002/jbt.2570060202 1941903 10.1002/jbt.2570060202

[CR54] Verhille M, Hausler R (2024) Evaluation of the impact of L-tryptophan on the toxicology of perfluorooctanoic acid in *Daphnia magna*: characterization and perspectives. Chemosphere 367:143665. 10.1016/j.chemosphere.2024.143665 39489306 10.1016/j.chemosphere.2024.143665

[CR55] von der Ohe PC, Liess M (2004) Relative sensitivity distribution of aquatic invertebrates to organic and metal compounds. Environ Toxicol Chem 23:150–156. 10.1897/02-577 14768879 10.1897/02-577

[CR56] Wang R, Zhang J, Yang Y, Chen CE, Zhang D, Tang J (2022) Emerging and legacy per- and polyfluoroalkyl substances in the rivers of a typical industrialized province of China: spatiotemporal variations, mass discharges and ecological risks. Front Environ Sci 10:986719. 10.3389/fenvs.2022.986719

[CR57] Wee SY, Aris AZ (2023) Revisiting the forever chemicals, PFOA and PFOS exposure in drinking water. NPJ Clean Water 6:57. 10.1038/s41545-023-00234-2

[CR58] Xie G, van Gestel CA, Vonk JA, Kraak MH (2025) Multigeneration responses of *Daphnia magna* to short-chain per- and polyfluorinated substances (PFAS). Ecotoxicol Environ Saf 294:118078. 10.1016/j.ecoenv.2025.118078 40120482 10.1016/j.ecoenv.2025.118078

[CR59] Yang HB, Zhao YZ, Tang Y, Gong HQ, Guo F, Sun WH, Liu SS, Tan H, Chen F (2019) Antioxidant defence system is responsible for the toxicological interactions of mixtures: A case study on PFOS and PFOA in *Daphnia magna*. Sci Total Environ 667:435–443. 10.1016/j.scitotenv.2019.02.378 30833242 10.1016/j.scitotenv.2019.02.418

[CR61] Zamora-Barrios CA, Nandini S, Sarma SSS (2024) Effect of microplastics on the demography of *Brachionus calyciflorus* Pallas (Rotifera) over successive generations. Aquat Toxicol 275:107061. 10.1016/j.aquatox.2024.107061 39217789 10.1016/j.aquatox.2024.107061

[CR63] Zamora-Barrios CA, Nandini S, Sarma SSS (2026) Combined effect of crude extracts from a cyanobacteria consortium and temperature on the demographic characteristics of *Ceriodaphnia dubia* (Cladocera). Limnology 1–18. 10.1007/s10201-026-00827-x

[CR62] Zamora-Barrios CA, Rodríguez MEF, Nandini S, Sarma SSS (2025) Comparative ecotoxicological effects of cyanobacterial crude extracts on native tropical cladocerans and *Daphnia magna*. Toxins 17:277. 10.3390/toxins17060277 40559855 10.3390/toxins17060277 PMC12197419

[CR64] Zhang L, Niu J, Li Y, Wang Y, Sun D (2013) Evaluating the sub-lethal toxicity of PFOS and PFOA using rotifer *Brachionus calyciflorus*. Environ Pollut 180:34–40. 10.1016/j.envpol.2013.04.010 23727565 10.1016/j.envpol.2013.04.031

[CR65] Zhang L, Niu J, Wang Y, Shi J, Huang Q (2014) Chronic effects of PFOA and PFOS on sexual reproduction of freshwater rotifer *Brachionus calyciflorus*. Chemosphere 114:114–120. 10.1016/j.chemosphere.2014.04.009 25113191 10.1016/j.chemosphere.2014.03.099

