# Supplementary material for: Multigenerational toxicity of perfluorooctanoic acid (PFOA) on the demography of Simocephalus vetulus (Branchiopoda)
Source: Ecotoxicology. 2026 Apr 24;35(5):104. doi: 10.1007/s10646-026-03079-8 (PMC13109218; doi:10.1007/s10646-026-03079-8)
Supplement: Supplementary file 1 — Supplementary Material 1 [file 10646_2026_3079_MOESM1_ESM.docx]

**Figure S1.** Matrix-matched calibration curves used for PFOA quantification: a) low-concentration linear range from 0.01 to 0.4 mg L^-1^, and b) high-concentration linear range from 0.5 to 4.0 mg L^-1^.

**Table S1.** Nominal (theoretical) and analytically measured PFOA concentrations used in the acute toxicity (LC50) assay. Concentrations were determined by HPLC–ESI–MS/MS in freshly prepared exposure media (t = 0 h) prior to organism exposure.

| **Sample** | **C_THEORETICAL_ (µg L^-1^)** | **C_ACTUAL_ (µg L^-1^)** |
| --- | --- | --- |
| Blank | - | - |
| LC001 | 100 | 108.16 |
| LC002 | 200 | 208.36 |
| LC003 | 400 | 404.17 |
| LC004 | 800 | 802.62 |
| LC005 | 1600 | 1632.48 |
| LC006 | 3200 | 3289.61 |

**Table S2.** Comparison between nominal (theoretical) and measured PFOA concentrations in freshly prepared exposure media for the chronic toxicity assays. Concentrations were determined by HPLC–ESI–MS/MS at t = 0 h, prior to organism exposure.

| **Sample** | **C_THEORETICAL_ (µg L^-1^)** | **C_ACTUAL_ (µg L^-1^)** |
| --- | --- | --- |
| Blank | - | - |
| C001 | 44 | 44.99 |
| C002 | 89 | 93.31 |
| C003 | 178 | 178.55 |
